# Predicting tumor dynamics in treated patients from patient-derived-xenograft mouse models: a translational model-based approach

**DOI:** 10.1007/s10928-025-09970-x

**Published:** 2025-04-16

**Authors:** D. Ronchi, E. M. Tosca, P. Magni

**Affiliations:** Dipartimento di Ingegneria Industriale e dell’Informazione, Università degli Studi di Pavia, 27100 Pavia, Italy

**Keywords:** Translational modeling, Tumor size dynamics, Patient-derived xenograft, Anticancer treatment prediction, Allometric scaling

## Abstract

This study presents a translational modeling framework designed to predict tumor size dynamics in cancer patients undergoing anticancer treatment, using data from patient-derived xenograft (PDX) mice. In the first step, a population tumor growth inhibition (TGI) model to estimate the distribution of exponential tumor growth rates and anticancer drug potency in PDX mice was built. Then, model parameters were allometrically scaled from mice to humans to inform a TGI model predicting tumor size dynamics in cancer patients. Longitudinal tumor dynamics predicted by the PDX-informed TGI model were expressed in terms of tumor progression events to allow validation against literature time-to-progression (TTP) data. The proposed approach was tested on two case studies: gemcitabine treatment for pancreatic cancer and sorafenib treatment for hepatocellular cancer. The framework successfully predicted median tumor size dynamics, closely aligned with clinical TTP curves for gemcitabine-pancreatic cancer case study. While predictions for extreme tumor size percentiles highlighted potential avenues for refinement, such as incorporating resistance mechanisms, the overall accuracy underscored the goodness of the approach. For the sorafenib-hepatocellular cancer case study, the framework provided plausible tumor size predictions, with TTP curves closely aligned with clinical observations, despite the limited availability of clinical data prevented a full validation. Overall, the translational modeling framework showed potential for predicting tumor dynamics in cancer patients, with results suggesting its applicability as a valid tool to support early decision-making in oncology.

**Supplementary Information:**

The online version contains supplementary material available at 10.1007/s10928-025-09970-x.

## Introduction

Assessment of tumor dynamics, defined as changes in tumor burden over time, is a critical component in evaluating the efficacy of cancer therapeutics during clinical trials [1]. Tumor dynamics not only reflect the immediate effect of treatments, such as tumor shrinkage or slowed growth, but also act as a proxy for predicting long-term outcomes, including overall survival (OS) [2, 3]. Consequently, they represent an essential early marker of efficacy in oncology drug development. Traditionally, tumor dynamics are monitored through the sum of the longest diameters (SLDs) of target lesions, as measured by imaging techniques and standardized by Response Evaluation Criteria in Solid Tumors (RECIST) [4, 5]. RECIST enables the classification of tumor dynamics into derived endpoints, such as objective response, which measures tumor shrinkage, and progression disease (PD), which captures tumor growth or the appearance of new lesions. These metrics are further summarized into clinical endpoints like objective response rate or time-to-progression (TTP), which are widely used in early-phase trials to assess treatment efficacy and guide the design of late-stage clinical studies [6].

Predicting tumor dynamics in advance holds significant value for oncology drug development, providing a preliminary assessment of treatment efficacy that can streamline clinical trial designs and improve decision-making processes. Preclinical studies in relevant in vivo models, such as patient-derived xenografts (PDXs), offer a rich source of information about treatment efficacy. These studies can be used to complement clinical evidence and support early clinical evaluations [7–9]. However, translating preclinical findings into clinical outcomes remains a significant challenge due to differences in biological contexts, variability in drug effects, and the inherent limitations of preclinical models [10, 11].

Mathematical modeling has shown great promise in bridging this gap, facilitating the translation of preclinical evidence into clinically relevant predictions. A multitude of mathematical models have been developed to describe tumor dynamics and its inhibition following anticancer drug treatment in xenograft mice [12–18]. These tumor growth inhibition (TGI) models have been used to develop preclinical-to-clinical translational approaches that can anticipate effective drug exposures in humans [19–23] or to predict longitudinal tumor size dynamics [24, 25].

Recently, a translational modeling framework was proposed to predict tumor dynamics in untreated cancer patients using tumor growth data from PDX mice [26]. This framework relied on the assumption that human solid tumors grow exponentially and that growth rates scale allometrically from PDX mice to humans. The approach was successfully applied to eleven different types of solid cancers and validated based on clinically observed tumor volume doubling times.

This work aims to extend the previous translational modeling framework incorporating the effect of an anticancer treatment. The objective is to predict expected tumor size dynamics in patients undergoing treatment, leveraging data collected during TGI studies in PDX mice. The proposed approach is here applied on two case studies (i.e., the gemcitabine treatment of pancreatic cancer and the sorafenib treatment of hepatocellular cancer), selected based on the availability of preclinical data. In both the cases, population-level predictions [27] of tumor dynamics were derived and validated against clinical TTP data, addressing the challenge of the absence of raw longitudinal tumor dynamics in treated patients. However, beyond the specific application here reported, the proposed framework is designed as a general approach, as it does not rely on case-specific assumptions, making it applicable to different solid cancers and anticancer treatments.

## Materials and methods

Starting from [26], a translational modeling framework was developed to predict tumor size dynamics in cancer patients undergoing specific anticancer treatments from TGI data in PDX mice.

**Fig. 1:**
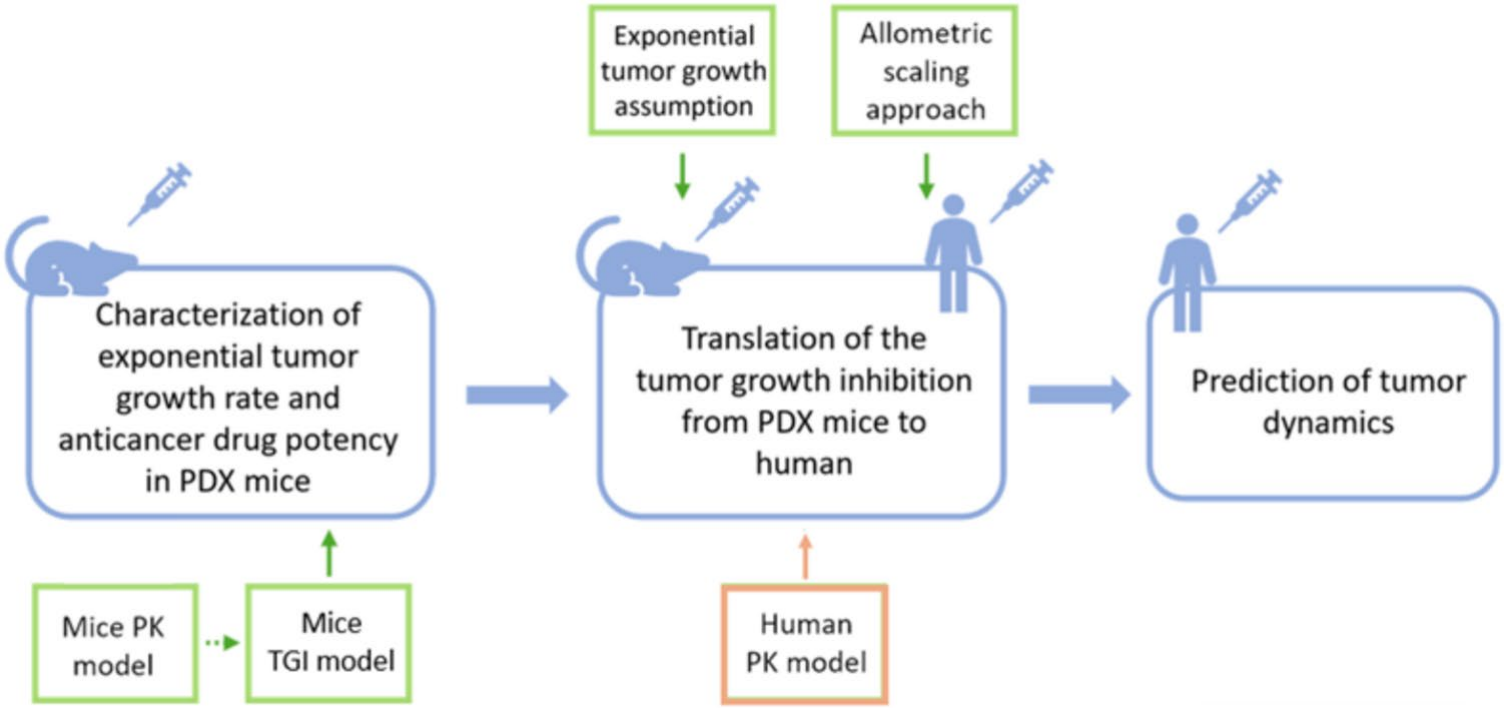
Flow chart representing the translational modeling approach

The approach, illustrated in Fig. 1, was based on a multistep procedure. First, for each case study, a population TGI model was developed to characterize the distribution of the exponential tumor growth rate and the anticancer drug potency in PDX mice. Second, these parameters were allometrically scaled from mice to humans and used to inform a TGI model predicting tumor size dynamics in cancer patients under anticancer drug treatment.

### Data

#### TGI data in PDX mice

Two panels of PDX mouse models for pancreatic (n = 27) and hepatocellular (n = 24) cancer were derived from HuBase database (Crownbio Bioscience Inc., https://www.crownbio.com/). For each PDX mouse model, a single TGI study composed of a control and a treated arm (both involving several mice) was selected. For pancreatic cancer, treated mice received gemcitabine administered intraperitoneally (i.p.) or intravenously (i.v.) with different protocols. For hepatocellular cancer, sorafenib was administered orally (o.s.) to mice at different doses and schedules. For each TGI study, average tumor weights in control and treated arms were made available.

The complete list of analyzed experiments with information on the administration protocols can be found in the *TGI data in PDX mice* Section of Supplementary Material S1.

#### Clinical data used to assess model predictions

Longitudinal tumor data in pancreatic and hepatocellular patients receiving gemcitabine or sorafenib, respectively, were searched to validate tumor dynamic predictions. However, as suitable raw longitudinal tumor data were not available, TTP data were utilized instead. Indeed, as TTP is defined as the time elapsed between treatment initiation and objective tumor progression [6], it provides an appropriate measure for evaluating the predictions of tumor dynamics.

For each case study, comprehensive literature search was conducted to retrieve suitable tumor progression data. Kaplan–Meier (KM) plots from published clinical studies were digitized, and median TTP values, defined as the time ${t}^{*}$ at which $TTP\left({t}^{*}\right)=0.5$, were extracted. The 90% confidence intervals (90%CI) for these values were also reconstructed.

When TTP data were not directly available from the reference clinical trials, progression events and TTP curves were reconstructed from reported Progression Free Survival (PFS) and OS. To this end, the relationships between PFS, OS, and TTP were leveraged, following the methodology described in [28] and detailed in the Supplementary Material S2. Selected references for the two case studies were listed in Tables 1 and 2, respectively.

**Table 1:** Selected TTP data for the gemcitabine treatment of the pancreatic cancer

Reference	Number of patients in the gemcitabine group	Median [90%CI] TTP time [months]
Hong [29]	56	3.9 [3.2–6.7]
Kindler [30]	316	7.5 [6.2–7.8]
Nakai [31]	53	4.7 [2.2–6.6]
Ozaka [32]	59	5.6 [3.3–7.1]

**Table 2:** Selected TTP data for the sorafenib treatment of the hepatocellular cancer

Reference	Number of patients in the sorafenib group	Median [90%CI] TTP time [months]
Park [33]	169	3.6 [3.1–3.8]
Qin [34]	331	3.6 [3.1–3.7]
Kudo [35]	103	3.5 [2.7–4.3]
Lyu [36]	132	4.3 [3.7–4.9]

For pancreatic cancer, in all the selected studies (Table 1) patients were treated following the nominal standardized protocol for gemcitabine monotherapy: weekly 30 min i.v. infusion of $1000\frac{mg}{{m}^{2}}$ for 7 weeks (or until unacceptable toxicity), followed by a 1 week rest period; subsequent cycles involved weekly infusions for 3 consecutive weeks out of every 4 [37].

For hepatocellular cancer, ethnicity resulted a significant covariate for both tumor growth rate and response to sorafenib treatment [38, 39]. Since all the considered PDX mouse models were derived from Asian patients (see Supplementary Material S1), the analysis was limited to clinical trials involving only Asian patients. Selected studies are listed in Table 2. In all of them, the nominal protocol involved 400 mg of sorafenib (two 200 mg tablets) taken orally twice daily, amounting to a total daily dose of 800 mg. Treatment should be maintained until either a clinical benefit is evident or unacceptable levels of toxicity are reached [40].

In addition to TTP data, for the gemcitabine treatment of pancreatic cancer case study, the TGI model for the SLDs developed by Garcia-Cremades et al. [41] provided a reference for clinical tumor size dynamics. Instead, for sorafenib treatment of hepatocellular cancer, no adequate clinical data or models for longitudinal tumor dynamics were found.

### PK/PD modelling of TGI in PDX mice

For each case study, the PK/PD Simeoni TGI model [13] was used to analyze TGI data in PDX mice. The model describes the temporal dynamics of tumor volume in control and treated mice. Specifically, in absence of treatment, the model assumes an exponential tumor growth pattern followed by a linear one, which are determined by the rates ${\lambda }_{0}$ [1/day] and ${\lambda }_{1}$ [cm^3^/day], respectively. In treated animals, the model assumes that a fraction of cells, hit by the drug, becomes not proliferating and progresses to death through a sequence of three damage stages according to the first-order constant ${k}_{1}$ [1/day]. Anticancer drug effect is governed by plasma concentration through the ${k}_{2}$ [L/mg∙day] parameter representing the drug potency.

Drug concentration profiles in input to the Simeoni TGI model were simulated with PK models derived from the literature. In particular, for gemcitabine i.v. the two-compartment model with linear elimination reported in [21] was used. For i.p. administration, a linear absorption compartment was added and absorption rate was identified on data derived from [42]. For sorafenib, the two-compartmental PK model developed by Choi et al. was selected [43]. For both the case studies, details about the PK models and the parameter values are reported in Supplementary Material S3.

For each case study, a population non-linear mixed-effects (NLME) approach was adopted to concurrently analyze data from all the PDX studies. All the model parameters were supposed to be lognormally distributed and parametrized as ${p}_{i}={\theta }_{p}{e}^{{\eta }_{{p}_{i}}}$ where ${\theta }_{p}$ represented the typical value and ${\eta }_{p} \sim N(0,{\omega }_{p}^{2})$ the random effect accounting for inter-PDX variability. Correlation was considered only between random effects of ${\lambda }_{0}$ and ${k}_{2}.$ Model fitting was performed adopting a combined residual error model for the gemcitabine case study and a proportional residual error model for the sorafenib one.

### PK/PD modeling of tumor response in human cancer patients

#### Human TGI model

Tumor mass in humans was assumed to grow exponentially with a rate ${\lambda }_{0,human}$. Drug was supposed to exert a direct anticancer effect on the exponential tumor growth rate, that is proportional to plasma drug concentration, $c\left(t\right),$ through the anticancer drug potency, ${k}_{2,human}.$ The dynamics of tumor volume in humans, $TV(t)$, are, thus, described by:

1 $$\frac{dTV\left( t \right)}{{dt}} = \left( {\lambda_{0,human} - k_{2,human} \cdot c\left( t \right)} \right) \cdot TV\left( t \right) {\text{  }} {\text{  }} {\text{   with   }} {\text{  }} TV\left( 0 \right) = TV_{0}$$

As tumor diameter is generally used as a measure of structural tumor changes in the clinical settings, the model-predicted tumor volume $TV(t)$ was converted into the corresponding tumor diameter $TD\left(t\right)$ using the formula for a sphere (Eq. 2):

2 $$TD\left( t \right) = 2 \cdot \sqrt[3]{{\frac{3 \cdot TV\left( t \right)}{{4\pi }}}}$$

Drug concentration profiles were simulated using human population PK models available from the literature. For gemcitabine, the population PK model developed by Zhang et al. [44] was selected. It is given by a two compartment-model for i.v. administration. For sorafenib, the PK model developed by Jain et al. [45] was used. It is composed by a central compartment and a four-compartment transition chain to account for absorption delay. Additionally, a semi-mechanistic model is integrated to depict the recirculation of the drug within the intestinal tract, known as enteropathic circulation. For both the drugs, concentration profiles were simulated using the typical model parameters.

#### Allometric scaling of exponential growth rate and anticancer drug potency from mice to humans

The exponential tumor growth rate ${\lambda }_{0}$ and the drug potency ${k}_{2}$ in PDX mice were scaled up to humans. Following the strategy proposed in [26], an allometric scaling approach was applied to both the parameters (Eqs. 3 and 4):

3 $$\lambda_{0,human} { }\left[ {\frac{1}{{{\text{months}}}}} \right] = 30 \cdot \lambda_{0,mouse} \cdot \left( {\frac{{BW_{human} }}{{BW_{mouse} }}} \right)^{ - \alpha }$$

4 $$k_{2,human} \left[ {\frac{{{\raise0.7ex\hbox{$L$} \!\mathord{\left/ {\vphantom {L g}}\right.\kern-0pt} \!\lower0.7ex\hbox{$g$}}}}{{{\text{months}}}}} \right] = 30 \cdot k_{2,mouse} \cdot \left( {\frac{{BW_{human} }}{{BW_{mouse} }}} \right)^{ - \alpha } \cdot \frac{{f_{u,human} }}{{f_{u,mouse} }}$$

where ${BW}_{human}$ and ${BW}_{mouse}$ represented the standard body weight of humans (70 kg) and mice (0.025 kg), respectively, $\alpha$ (= 1/3) was the allometric exponent, 30 was the unit conversion factor from day to month and ${f}_{u,human}$ and ${f}_{u,mouse}$ represent the drug-specific unbound fraction in human and mouse, respectively.

For both the case studies, a multivariate lognormal distribution for ${\lambda }_{0,\text{human}}$ and ${k}_{2,human}$ was predicted propagating the inter-PDX variability. Distribution was parametrized as

5 $$\left(\begin{array}{c}{\lambda }_{0,human,i}\\ {k}_{2,human,i}\end{array}\right) = \left(\begin{array}{c}{\lambda }_{0,human,pop}\\ {k}_{2,human,pop}\end{array}\right){e}^{{\overline{\eta }}_{human,i}}$$

where $\lambda_{0,human,pop} = 30 \cdot \lambda_{0,pop} \cdot \left( {BW_{human} /BW_{mouse} } \right)^{ - \alpha }$ and $k_{2,human,pop} = 30 \cdot k_{2,pop} \cdot \left( {BW_{human} /BW_{mouse} } \right)^{ - \alpha } \cdot f_{u,human} /f_{u,mouse}$ are the typical values and ${{\overline{\eta }}_{human}=\left({\eta }_{{\lambda }_{0,human}},{\eta }_{{k}_{2,human}}\right) }^{T}\sim N(0,{\Omega }_{human})$ with $${\Omega }_{human}=\left(\begin{array}{cc}{\omega }_{{\lambda }_{0}}^{2}& {\omega }_{{\lambda }_{0},{k}_{2}}\\ {\omega }_{{\lambda }_{0},{k}_{2}}& {\omega }_{{k}_{2}}^{2}\end{array}\right)$$ a submatrix of ${\Omega }_{mouse}$.

### Prediction and validation of tumor dynamics in treated cancer patients

For each case study, 200 virtual cancer patients were generated, each characterized by a set of individual parameters of the human TGI model, i.e., (${\lambda }_{0,human,i}; {k}_{2,human,i}$), which were sampled from the scaled multivariate lognormal distribution (Eq. 5). Given the behavior of the translated TGI model which assumes an exponential tumor growth combined with a first order drug effect, the predicted times to tumor progression are independent of initial tumor dimensions. Thus, $T{V}_{0}$ was fixed at 1 cm^3^. For the gemcitabine/pancreatic cancer case study, only to enable a direct comparison between the longitudinal tumor profiles predicted by our translational approach and those simulated by the TGI model developed by Garcia et al. on clinical data, we adopted the same baseline tumor size distributions as those reported in Garcia’s publication [41]. For each virtual cancer patient, the dynamics of tumor diameter were simulated assuming strict adherence to the treatment protocol without any modifications or interruptions throughout the entire simulation time.

To compute TTP, for each virtual patient, the time to progression was derived from the predicted tumor dynamics. To this end, simplifying RECIST definition, only PD events caused by a 20% increase of SLD of pre-identified target lesions were considered. Then, the KM curve for the 200 virtual patients was constructed.

A Monte Carlo simulation framework, accounting for parameter estimation uncertainty, was employed to enhance the generalizability and robustness of predictions. To this end, the previous procedure was repeated 1000 times, i.e., 1000 in silico trials in 1000 virtual patient cohorts each composed by 200 individuals were performed. Medians and 90% prediction intervals (90%PIs) across the 1000 replicates were, then, computed for both tumor diameter trajectory and TTP curve. Details on the simulation procedure were reported in the Supplementary Material S4.

### Software

The population Simeoni TGI models were identified using Monolix 2021R2. Simulations were conducted using the ‘*lixoftConnectors’* R package within RStudio, after importing the estimated models into Simulx 2021R2.

## Results

### Gemcitabine treatment of pancreatic cancer

The population Simeoni TGI model was successfully identified on data from the 27 PDX studies available for gemcitabine and the distributions of model parameters were derived (results reported in the Supplementary Material S5). Estimated ${\lambda }_{0}$ and ${k}_{2}$ values were scaled up from mice to humans according to the allometric rules reported in Eqs. 3 and 4. In particular, for ${k}_{2}$ the $\frac{{f}_{u,human}}{{f}_{u,mouse}}$ ratio was set to 1, as no significant differences were found between unbound fractions of gemcitabine in mice and humans [37, 46].

**Fig. 2:**
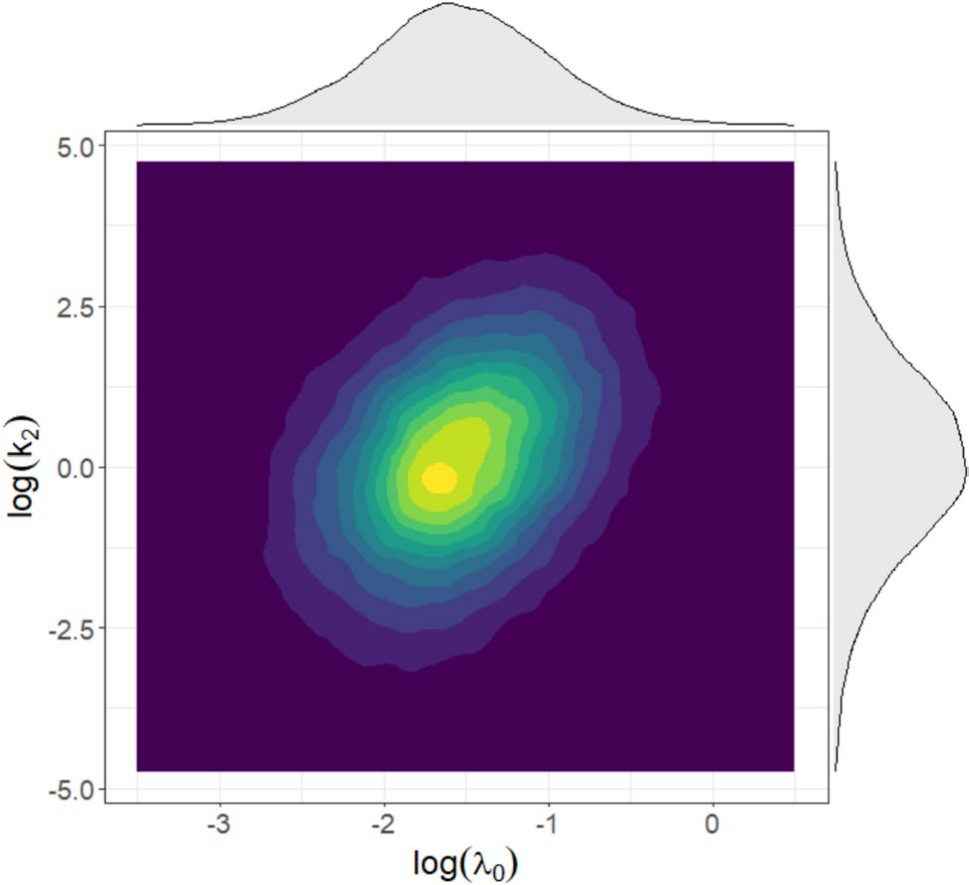
Log-transformed marginal distributions of the ${\uplambda }_{0}$ and ${\text{k}}_{2}$ for the generated pancreatic cancer virtual patients treated with gemcitabine

**Fig. 3:**
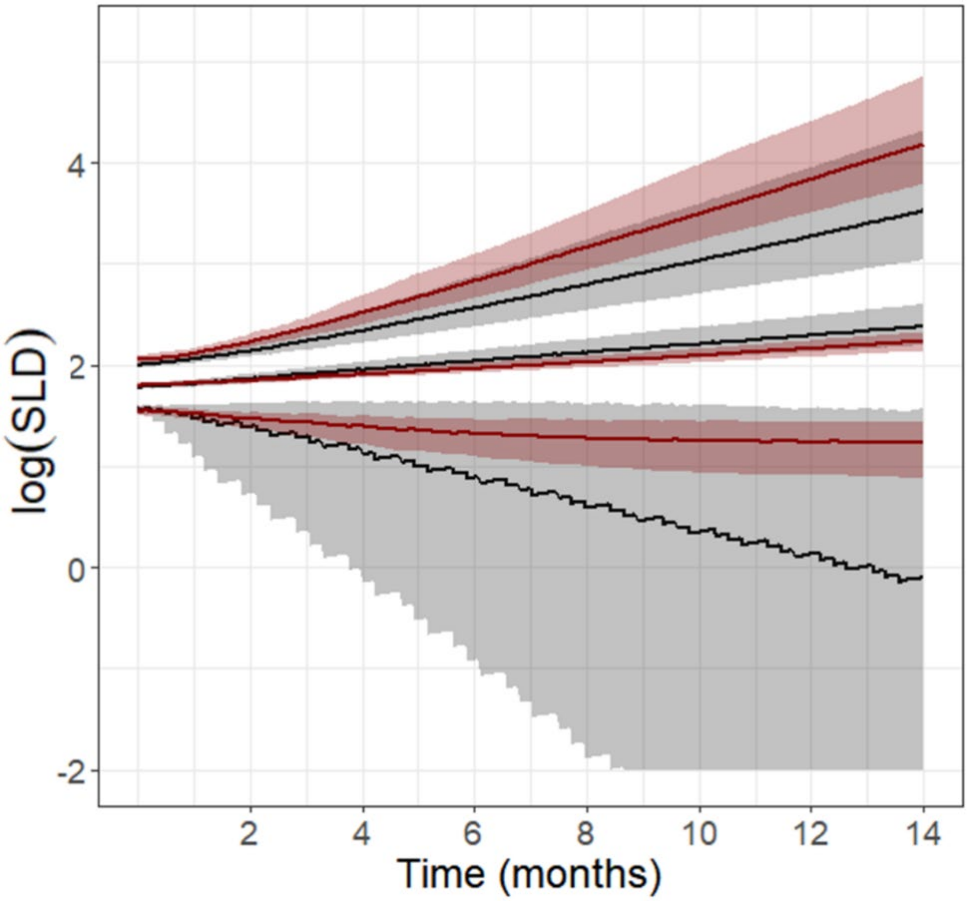
VPC of the simulated SLD trajectories over a 14 months period under the standard gemcitabine treatment schedule. Simulated tumor dynamics predicted from PDX mice data and by the Garcia-Cremades TGI model [41] were reported in black and red, respectively. Solid lines represent the median and the 5th and 95th percentiles; dashed areas the corresponding 90%PI (Color figure online)

1000 virtual patient cohorts, each composed of 200 individuals, were generated (Fig. 2). To enable a direct comparison, the initial tumor size distribution reported in [41] was used, converting baseline SLD values into TV under the assumption of spherical shape: $T{V}_{0,i} [{\text{cm}}^{3}]$=115.3 ${e}^{{{\eta }_{TV}}_{i}}$ where ${{\eta }_{TV}}_{i}\sim N(\text{0,0.16}$). In addition, the same simulation procedure described in the “Prediction and validation of tumor dynamics in treated cancer patients” section was applied also for simulations of the Garcia-Cremades TGI model. The comparison highlights a notable overlap between the curves (Fig. 3).

Overall, tumor profiles predicted from PDX data were in good agreement with the SLD trajectories simulated using the reference clinical model. In particular, the two median tumor profiles resulted very close for the entire simulated period, showcasing nearly complete alignment between the model informed by the PDX studies and the one developed based on clinical data. However, considering the 5th and 95th percentiles, the model informed by the PDX data predicted a greater anticancer effect for gemcitabine or a slower tumor growth respect to the clinical model. This discrepancy was probably due to the presence of a drug resistance mechanism, resulting in a diminished effect after 2 months, that was incorporated in the model developed by Garcia et al. and not considered in the TGI model scaled from PDX mice.

**Fig. 4:**
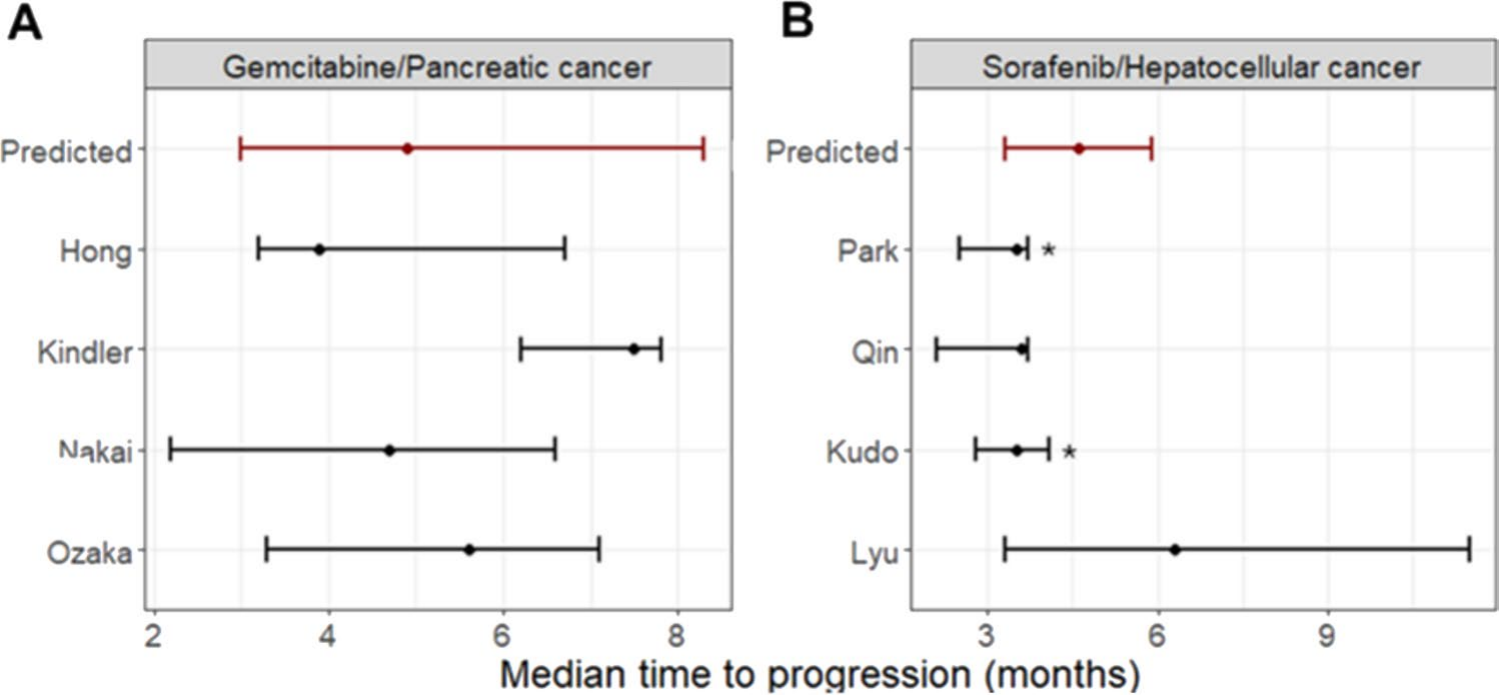
Median TTP for the two case studies: pancreatic cancer treated with gemcitabine (Panel **A**) and hepatocellular cancer treated with sorafenib (Panel **B**). Black dots indicate the median TTP with black error bars indicating their 90%CI, respectively. Red dots and error bars indicate the predicted medians TTP and the 90%PI, respectively. * marks studies for which digitized TTP data were directly used (Color figure online)

**Fig. 5:**
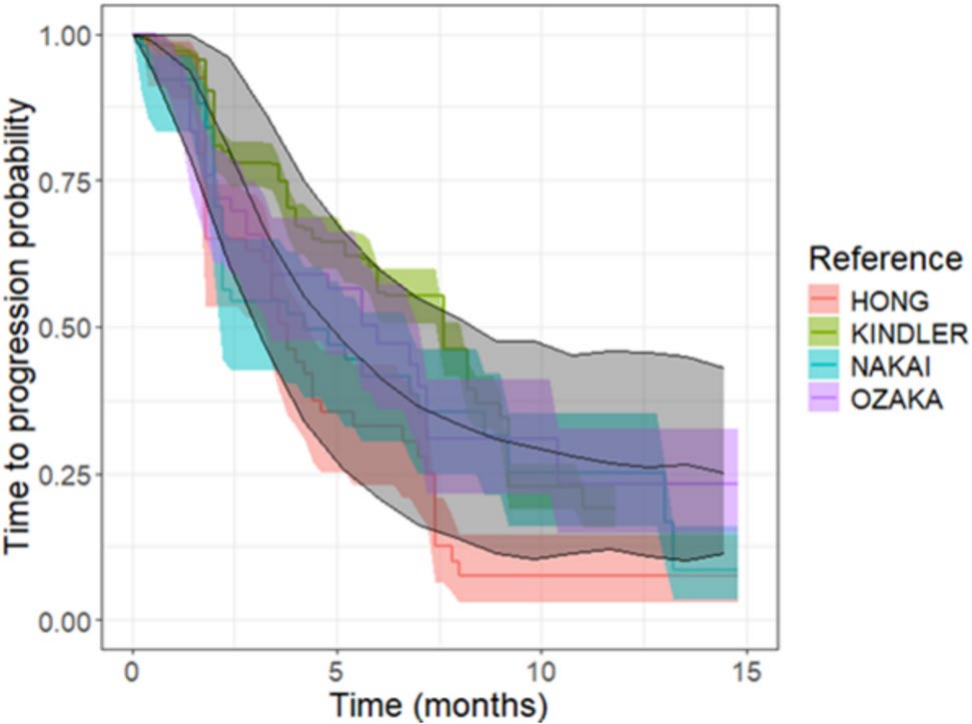
KM-VPC plots (grey areas) generated from 1000 virtual clinical trials composed of 200 individuals with pancreatic cancer treated the standard gemcitabine schedule were superimposed to TTP curves derived from literature studies (colored areas). The gray line and shaded area represent the median and 90%PI from the virtual trials, while the colored lines and areas depict the medians and 90%CI from the literature studies (Color figure online)

In terms of TTP, the translational approach predicted a median time to tumor progression of 4.9 months, with a 90%PI between 2.6 and 8.5 months. The median TTP from all the reference studies fell within the 90%PI (Table 1, Fig. 4A). In addition, the overall shape of the TTP curve (Fig. 5) closely aligned with clinical observations.

### Sorafenib treatment of hepatocellular cancer

As for the gemcitabine case study, a population model based on the Simeoni TGI model was developed on data from PDX studies involving sorafenib treatment (Supplementary Material S5). The estimated ${\lambda }_{0}$ and ${k}_{2}$ parameters were then allometrically scaled to humans (Eqs. 4 and 5), assuming that the unbound sorafenib fraction was the same for mice and humans [47].

**Fig. 6:**
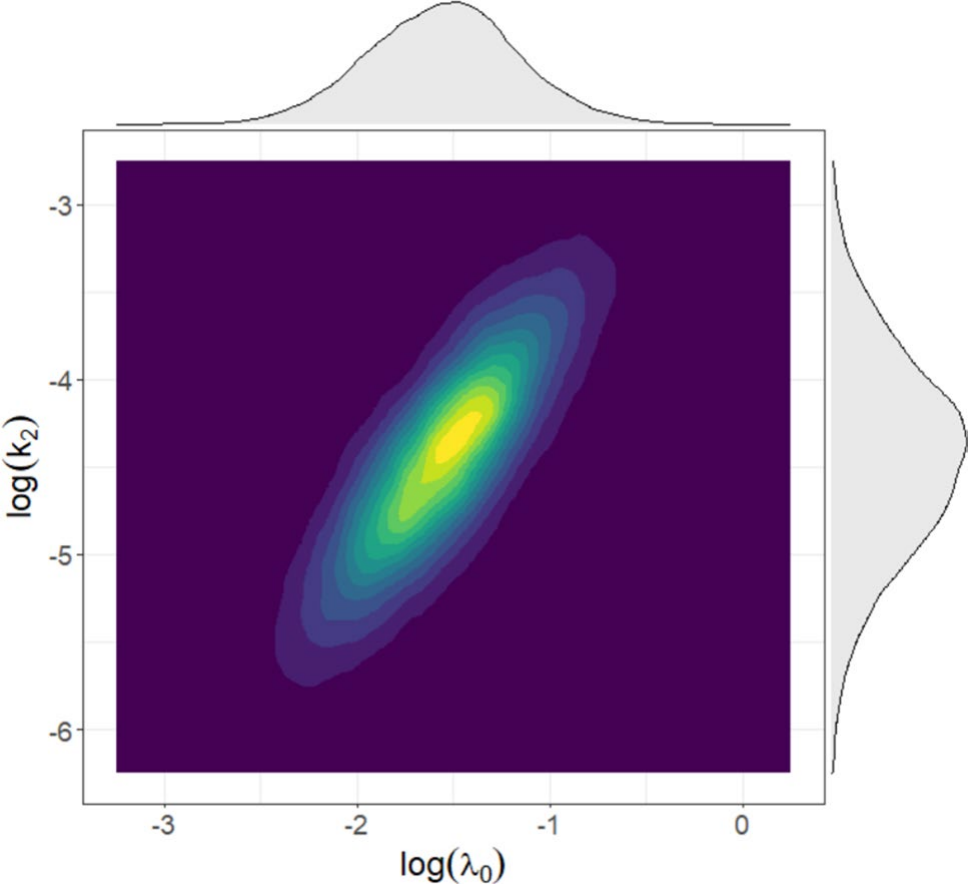
Log-transformed marginal distributions of the ${\uplambda }_{0}$ and ${\text{k}}_{2}$ for the generated hepatocellular cancer virtual patients treated with sorafenib

One thousand virtual patient cohorts, each composed of 200 individuals (Fig. 6), were generated and tumor dynamics predicted assuming the arbitrary baseline tumor dimensions of $1 {\text{cm}}^{3}$ (Fig. S4 of Supplementary Material S6). Unfortunately, for this case study adequate clinical data or mathematical models for predicting longitudinal tumor dynamics to use as reference were not available.

**Fig. 7:**
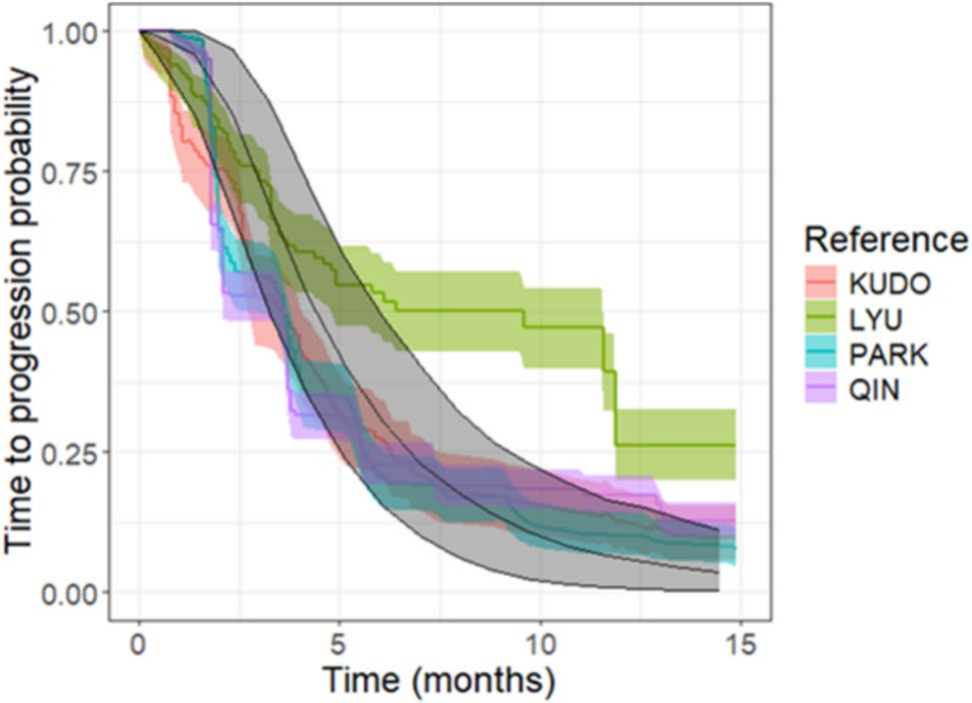
KM-VPC plots (grey areas) generated from 1000 virtual clinical trials composed of 200 individuals with hepatocellular cancer treated the standard sorafenib schedule were superimposed to TTP curves derived from literature studies (colored areas). The gray line and shaded area represent the median and 90%PI from the virtual trials, while the colored lines and areas depict the medians and 90%CI from the literature studies (Color figure online)

In terms of TTP, the translational approach predicted a median time to progression of 4.7 months, with a 90%PI from 3.3 to 5.9 months. Despite a general overestimation, observed medians fell within the 90%PI for 3 out of 4 studies [33–35]. Lyu et al. [36] showed a completely different behaviour than the other 3 studies and its median TTP was not accurately captured (Fig. 4B). When analyzing the overall shape of the TTP curves, a slight underestimation of progression at early time points was observed for the three similar studies, while longer times to progression were well predicted. In contrast, the clinical trial data from Lyu et al. exhibited an opposite trend (Fig. 7).

## Discussions

In this work, a translational modeling framework to predict longitudinal tumor size dynamics in cancer patients undergoing an anticancer treatment from TGI data in PDX mice was proposed. To this end, a recent translational approach [26] was extended to incorporate the effects of anticancer therapies. The result is a multistep procedure that, from a theoretical point of view, can be applicable to a multitude of cancer types and anticancer treatments. This approach was here applied on two case studies, i.e., the gemcitabine treatment of pancreatic cancer and the sorafenib treatment of hepatocellular cancer. For each cancer type and anticancer treatment, first, a set of TGI studies in a panel of PDX mouse models was analyzed through a mathematical modeling approach to estimate the exponential tumor growth rate and the anticancer drug potency in PDX mice. The Simeoni TGI model was selected and combined with a population NLME approach. A multivariate log-normal distribution for the exponential tumor growth rate and anticancer drug potency (${{\lambda}}_{0}$ and ${{k}}_{2}$ parameters) was identified accounting for the inter-PDX variability. Second, parameters were scaled up from PDX mice to humans adopting an allometric strategy (Eqs. 3, 4). The inter-PDX variability was propagated, and a multivariate log-normal distribution was derived for, ${\lambda }_{0,human}-{k}_{2,human}$ (Eq. 5). Scaled parameters were used to inform a TGI model [48] predicting tumor size dynamics in cancer patients under anticancer drug treatment. An exponential tumor growth model incorporating a first order drug effect was assumed and combined with clinical PK models derived from the literature. Predictions of tumor progression dynamics were simulated with the scaled TGI model and validated against clinical TTP curves derived from the literature. For the case study relating to the gemcitabine treatment of pancreatic cancer, a panel of 27 TGI studies in PDX mouse model was considered. From that, the longitudinal tumor size dynamics in a population of virtual patients undergoing standard gemcitabine administration protocol were predicted. SLD profiles simulated with a TGI model directly developed on clinical data were used as benchmark against which comparing predictions. The scaled TGI model informed by PDX data demonstrated robust predictive capabilities, predicting a median SLD profile extremely close to the clinical one. Differently, more extreme percentiles, i.e., 5th and 95th percentiles, of clinical SLD profiles resulted under-predicted by the PDX-informed TGI model. This discrepancy could be due to different factors. For example, the model derived from the PDX data did not consider the mechanism of resistance to gemcitabine, which was instead incorporated into the clinical model. Further, the inter-individual variability affecting tumor growth and response to gemcitabine in cancer patients could not be fully grasped by the inter-PDX viability identified on limited number of PDX mouse model. A not-well approximated (exponential) growth of pancreatic cancer could not be a priori excluded. In terms of TTP, predicted tumor progression dynamics were in agreement with clinical evidence, because TTP curves generated by the PDX-informed TGI model closely aligned with those derived from clinical trials used as references.

Similarly, for sorafenib treatment of hepatocellular cancer, 24 TGI studies in PDX mice were analyzed with the Simeoni TGI model and parameter estimates used to inform the TGI model in humans. The predicted tumor size profiles in patients under standard sorafenib administration exhibited plausible dynamics. However, the lack of adequate clinical data or model prevented an actual assessment. After expressing the predicted tumor dynamics in terms of TTP and comparing them with TTP curves derived from the literature, it can be observed that predictions slightly underestimate tumor progression at early time points. This discrepancy might be due to the model limitations in fully capturing early-stage tumor responses or to biological differences between PDX mice and human patients. On the other hand, the model performed well in predicting longer times to progression in all the studies, with the only exception of TTP from Lyu’s reference. Of note, Lyu’s [36] study exhibits TTP dynamics distinct from the other three studies.

Overall, the translational modeling framework proposed in this work is able to adequately predict tumor dynamics in cancer patients undergoing anticancer treatments. However, some limitations should be taken into consideration.

First, a robust validation of the predicted tumor size dynamics is still missing. Indeed, due to the lack of adequate clinical data to be used as benchmark, model predictions were validated only against TTP data derived from the literature. Based on RECIST 1.1 [4] PD events include (i) at least a 20% increase in the SLD of target lesions, with an absolute increase of at least 5 mm, (ii) unequivocal progression of existing non-target lesions, or (iii) appearance of new lesions. Differently, in this work PD events were simply defined as a 20% increase in SLD of target lesions. Excluding non-target lesion progression could lead to an underestimation of PD events, potentially resulting in an overestimation of TTP. Conversely, omitting the 5 mm threshold for the absolute SLD increase of target lesions may result in an overestimation of PD events, which could, in turn, lead to an underestimation of TTP. Although this deviation from the RECIST 1.1 definition could influence the comparison between predicted and observed TTP curves, the information that can be translated from preclinical TGI studies in PDX mouse models to the clinical setting does not support a more sophisticated definition of PD events. Additional challenges arise from the assumption of strict adherence to the nominal treatment protocol. This could result in overestimating TTP, especially if deviations from standard dosing schedules are significant. Furthermore, the presence of censored events in the observed TTP curves, such as patients lost to follow-up or died without documented progression, could also affect the comparison and introduce variability. Another potential source of bias stems from the reconstruction of the TTP curves from observed PFS and OS data. While this method allows for comparisons in the absence of explicit TTP data, it relies on assumptions about the relationship between progression and survival that may oversimplify the complexities of tumor dynamics. This could introduce additional uncertainties, highlighting the need for caution when interpreting the reconstructed curves and their impact on the robustness of our validation.

Second, the TGI model adopted to describe tumor dynamics in humans [48] relied on some relevant assumptions, such as an exponential tumor growth, an anticancer effect proportional to plasma drug concentration and the representation of the overall tumor burden as a single spherical mass. While the exponential growth model is the most commonly adopted to describe tumor growth in the clinical settings [41, 49, 50], the presence of possible more complex dynamics could not be excluded. Regarding the assumption of proportionality for the anticancer effect, this was informed by the preclinical studies in PDX mouse models, where a linear relationship between drug concentration and effect was observed. However, if non-linear behaviors or some resistance mechanisms to treatment were evident, the modeling framework is flexible enough to incorporate these dynamics. Additionally, the representation of the overall tumor burden as a single spherical tumor mass was necessary to convert preclinical volume-based measurements to clinical SLD-based measurements. Although this approach is widely used in translational studies [25, 51], it does introduce limitations, particularly when dealing with tumors that have irregular shapes [52].

Finally, the proposed translational modeling framework intended to be a general approach applicable to different cancer types and anticancer treatments, as no case-specific assumptions were made. However, it has been assessed only in two case studies so far. A further validation of the approach across a wider range of panel of cancer types and anticancer treatments, including targeted therapies and immunotherapies, is needed to demonstrate generalizability of the obtained findings, even if no specific issues are expected. Current efforts focus on extending the application of the proposed approach to other case studies, with the goal of strengthening and demonstrating the versatility of the proposed model in a variety of clinical settings.

Once the previous limitations are addressed, this translational modeling approach might be applied for multiple purposes across various stages of drug development. For instance, it could be used to predict tumor dynamics in response to treatment based on preclinical experiments, offering early insights into the drug therapeutic potential during or even before first clinical studies. Indeed, although in this work the translational framework was integrated with clinical-derived PK models, these could potentially be replaced with allometry-based predictions of human PK. In the late clinical stages, the framework can assist in the development of TGI models on clinical data by providing prior information on parameter distributions. Additionally, it can be used to generate virtual patient cohorts undergoing standard-of-care (SOC) treatments exploiting available TGI studies in PDX mice. They could act as synthetic control arms in single-arm clinical trials, providing a valuable alternative to external arms derived from historical clinical trials [53–55]. Overall, this translational framework offers a versatile range of applications that can support various stages of oncology drug development, in line with the principles of model-informed drug.

## Conclusions

In this work, a translational modeling framework was proposed and tested. It was able to predict longitudinal tumor size dynamics in cancer patients under anticancer treatments starting only from TGI studies in PDX mice. The approach was here successfully validated on the gemcitabine treatment of pancreatic cancer and sorafenib treatment of hepatocellular carcinoma. Although the framework was applied to only two case studies, its design is based on general principles and does not rely on case-specific assumptions, highlighting its potential for broader applicability. Further validation across diverse cancer types and therapeutic modalities is necessary to establish its robustness and confirm its generalizability. Future research will focus on extending the application of this modeling approach to additional case studies and investigating its potential to enhance preclinical-to-clinical translation for a variety of anticancer agents.

## Supplementary Information

Below is the link to the electronic supplementary material.

Supplementary file1 (XLSX 45 kb)

Supplementary file2 (PDF 614 kb)

## Data Availability

Data and references are provided within the manuscript or supplementary information files.

## References

[1] Bruno R, Bottino D, de Alwis DP, Fojo AT, Guedj J, Liu C, Swanson KR, Zheng J, Zheng Y, Jin JY (2020) Progress and opportunities to advance clinical cancer therapeutics using tumor dynamic models. Clin Cancer Res Off J Am Assoc Cancer Res 26:1787–1795. 10.1158/1078-0432.CCR-19-0287 10.1158/1078-0432.CCR-19-0287 PMC8415106 31871299

[2] Cremolini C, Loupakis F, Antoniotti C, Lonardi S, Masi G, Salvatore L, Cortesi E, Tomasello G, Spadi R, Zaniboni A, Tonini G, Barone C, Vitello S, Longarini R, Bonetti A, D’Amico M, Di Donato S, Granetto C, Boni L, Falcone A (2015) Early tumor shrinkage and depth of response predict long-term outcome in metastatic colorectal cancer patients treated with first-line chemotherapy plus bevacizumab: results from phase III TRIBE trial by the Gruppo Oncologico del Nord Ovest. Ann Oncol Off J Eur Soc Med Oncol 26:1188–1194. 10.1093/annonc/mdv112 10.1093/annonc/mdv112 25712456

[3] Piessevaux H, Buyse M, Schlichting M, Van Cutsem E, Bokemeyer C, Heeger S, Tejpar S (2013) Use of early tumor shrinkage to predict long-term outcome in metastatic colorectal cancer treated with cetuximab. J Clin Oncol Off J Am Soc Clin Oncol 31:3764–3775. 10.1200/JCO.2012.42.8532 10.1200/JCO.2012.42.8532 24043732

[4] Eisenhauer EA, Therasse P, Bogaerts J, Schwartz LH, Sargent D, Ford R, Dancey J, Arbuck S, Gwyther S, Mooney M, Rubinstein L, Shankar L, Dodd L, Kaplan R, Lacombe D, Verweij J (2009) New response evaluation criteria in solid tumours: revised RECIST guideline (version 1.1). Eur J Cancer Oxf Engl 1990(45):228–247. 10.1016/j.ejca.2008.10.026 10.1016/j.ejca.2008.10.026 19097774

[5] Padhani AR, Ollivier L (2001) The RECIST (response evaluation criteria in solid tumors) criteria: implications for diagnostic radiologists. Br J Radiol 74:983–986. 10.1259/bjr.74.887.740983 11709461 10.1259/bjr.74.887.740983

[6] FDA (2018) Clinical trial endpoints for the approval of cancer drugs and biologics guidance for industry. https://www.fda.gov/media/71195/download

[7] Abdolahi S, Ghazvinian Z, Muhammadnejad S, Saleh M, Asadzadeh Aghdaei H, Baghaei K (2022) Patient-derived xenograft (PDX) models, applications and challenges in cancer research. J Transl Med 20:206. 10.1186/s12967-022-03405-8 35538576 10.1186/s12967-022-03405-8 PMC9088152

[8] Sajjad H, Imtiaz S, Noor T, Siddiqui YH, Sajjad A, Zia M (2021) Cancer models in preclinical research: a chronicle review of advancement in effective cancer research. Anim Models Exp Med 4:87–103. 10.1002/ame2.12165 10.1002/ame2.12165 PMC8212826 34179717

[9] Ireson CR, Alavijeh MS, Palmer AM, Fowler ER, Jones HJ (2019) The role of mouse tumour models in the discovery and development of anticancer drugs. Br J Cancer 121:101–108. 10.1038/s41416-019-0495-5 31231121 10.1038/s41416-019-0495-5 PMC6738037

[10] Yates JWT, Byrne H, Chapman SC, Chen T, Cucurull-Sanchez L, Delgado-SanMartin J, Di Veroli G, Dovedi SJ, Dunlop C, Jena R, Jodrell D, Martin E, Mercier F, Ramos-Montoya A, Struemper H, Vicini P (2020) Opportunities for quantitative translational modeling in oncology. Clin Pharmacol Ther 108:447–457. 10.1002/cpt.1963 32569424 10.1002/cpt.1963

[11] Pagano E, Bergamo A, Carpi S, Donnini S, Notarbartolo Di Villarosa M, Serpe L, Lisi L (2021) Preclinical models in oncological pharmacology: limits and advantages. Pharmadvances. 10.36118/pharmadvances.2021.05

[12] Carrara L, Lavezzi SM, Borella E, De Nicolao G, Magni P, Poggesi I (2017) Current mathematical models for cancer drug discovery. Expert Opin Drug Discov 12:785–799 28595492 10.1080/17460441.2017.1340271

[13] Simeoni M, Magni P, Cammia C, De Nicolao G, Croci V, Pesenti E, Germani M, Poggesi I, Rocchetti M (2004) Predictive pharmacokinetic-pharmacodynamic modeling of tumor growth kinetics in xenograft models after administration of anticancer agents. Cancer Res 64:1094–1101. 10.1158/0008-5472.can-03-2524 14871843 10.1158/0008-5472.can-03-2524

[14] Tosca EM, Rocchetti M, Pesenti E, Magni P (2020) A tumor-in-host DEB-based approach for modeling cachexia and bevacizumab resistance. Cancer Res 80:820–831. 10.1158/0008-5472.CAN-19-0811 31818849 10.1158/0008-5472.CAN-19-0811

[15] Terranova N, Tosca EM, Pesenti E, Rocchetti M, Magni P (2018) Modeling tumor growth inhibition and toxicity outcome after administration of anticancer agents in xenograft mice: a dynamic energy budget (DEB) approach. J Theor Biol 450:1–14 29680449 10.1016/j.jtbi.2018.04.012

[16] Tosca EM, Pigatto MC, Dalla Costa T, Magni P (2019) A population dynamic energy budget-based tumor growth inhibition model for etoposide effects on wistar rats. Pharm Res 36:38 30635794 10.1007/s11095-019-2568-9

[17] Tosca EM, Gauderat G, Fouliard S, Burbridge M, Chenel M, Magni P (2021) Modeling restoration of gefitinib efficacy by co-administration of MET inhibitors in an EGFR inhibitor-resistant NSCLC xenograft model: a tumor-in-host DEB-based approach. CPT Pharmacomet Syst Pharmacol. 10.1002/psp4.12710 10.1002/psp4.12710 PMC8592518 34708556

[18] Garcia-Cremades M, Pitou C, Iversen PW, Troconiz IF (2017) Characterizing gemcitabine effects administered as single agent or combined with carboplatin in mice pancreatic and ovarian cancer xenografts: a semimechanistic pharmacokinetic/pharmacodynamics tumor growth-response model. J Pharmacol Exp Ther 360:445–456. 10.1124/jpet.116.237610 28028124 10.1124/jpet.116.237610

[19] Tosca EM, Borella E, Piana C, Bouchene S, Merlino G, Fiascarelli A, Mazzei P, Magni P (2023) Model-based prediction of effective target exposure for MEN1611 in combination with trastuzumab in HER2-positive advanced or metastatic breast cancer patients. CPT Pharmacomet Syst Pharmacol 12:1626–1639. 10.1002/psp4.12910 10.1002/psp4.12910 PMC10681519 36793223

[20] Tosca EM, Terranova N, Stuyckens K, Dosne AG, Perera T, Vialard J, King P, Verhulst T, Perez-Ruixo JJ, Magni P, Poggesi I (2022) A translational model-based approach to inform the choice of the dose in phase 1 oncology trials: the case study of erdafitinib. Cancer Chemother Pharmacol 89:117–128. 10.1007/s00280-021-04370-7 34786600 10.1007/s00280-021-04370-7

[21] Rocchetti M, Simeoni M, Pesenti E, De Nicolao G, Poggesi I (2007) Predicting the active doses in humans from animal studies: a novel approach in oncology. Eur J Cancer Oxf Engl 1990(43):1862–1868. 10.1016/j.ejca.2007.05.011 10.1016/j.ejca.2007.05.011 17604156

[22] Wong H, Choo EF, Alicke B, Ding X, La H, McNamara E, Theil FP, Tibbitts J, Friedman LS, Hop CECA, Gould SE (2012) Antitumor activity of targeted and cytotoxic agents in murine subcutaneous tumor models correlates with clinical response. Clin Cancer Res. 10.1158/1078-0432.CCR-12-0738 22648270 10.1158/1078-0432.CCR-12-0738

[23] Wong H, Vernillet L, Peterson A, Ware JA, Lee L, Martini JF, Yu P, Li C, Del Rosario G, Choo EF, Hoeflich KP, Shi Y, Aftab BT, Aoyama R, Lam ST, Belvin M, Prescott J (2012) Bridging the gap between preclinical and clinical studies using pharmacokinetic-pharmacodynamic modeling: an analysis of GDC-0973, a MEK inhibitor. Clin Cancer Res. 10.1158/1078-0432.CCR-12-0445 22496205 10.1158/1078-0432.CCR-12-0445

[24] Garcia-Cremades M, Pitou C, Iversen PW, Troconiz IF (2019) Translational framework predicting tumour response in gemcitabine-treated patients with advanced pancreatic and ovarian cancer from xenograft studies. AAPS J 21:23. 10.1208/s12248-018-0291-9 30706160 10.1208/s12248-018-0291-9

[25] Baaz M, Cardilin T, Lignet F, Jirstrand M (2022) Optimized scaling of translational factors in oncology: from xenografts to RECIST. Cancer Chemother Pharmacol 90:239–250. 10.1007/s00280-022-04458-8 35922568 10.1007/s00280-022-04458-8 PMC9402719

[26] Tosca EM, Ronchi D, Rocchetti M, Magni P (2024) Predicting tumor volume doubling time and progression-free survival in untreated patients from patient-derived-xenograft (PDX) models: a translational model-based approach. AAPS J 26:92. 10.1208/s12248-024-00960-4 39117850 10.1208/s12248-024-00960-4

[27] Mistry HB (2017) On the relationship between tumour growth rate and survival in non-small cell lung cancer. PeerJ 5:e4111. 10.7717/peerj.4111 29201573 10.7717/peerj.4111 PMC5712205

[28] Nagase M, Doshi S, Dutta S, Lin C-W (2022) Estimation of time to progression and post progression survival using joint modeling of summary level OS and PFS data with an ordinary differential equation model. J Pharmacokinet Pharmacodyn 49:455–469. 10.1007/s10928-022-09816-w 35870059 10.1007/s10928-022-09816-w

[29] Hong JY, Nam EM, Lee J, Park JO, Lee S-C, Song S-Y, Choi SH, Heo JS, Park SH, Lim HY, Kang WK, Park YS (2014) Randomized double-blinded, placebo-controlled phase II trial of simvastatin and gemcitabine in advanced pancreatic cancer patients. Cancer Chemother Pharmacol 73:125–130. 10.1007/s00280-013-2328-1 24162380 10.1007/s00280-013-2328-1

[30] Kindler HL, Ioka T, Richel DJ, Bennouna J, Létourneau R, Okusaka T, Funakoshi A, Furuse J, Park YS, Ohkawa S, Springett GM, Wasan HS, Trask PC, Bycott P, Ricart AD, Kim S, Van Cutsem E (2011) Axitinib plus gemcitabine versus placebo plus gemcitabine in patients with advanced pancreatic adenocarcinoma: a double-blind randomised phase 3 study. Lancet Oncol 12:256–262. 10.1016/S1470-2045(11)70004-3 21306953 10.1016/S1470-2045(11)70004-3

[31] Nakai Y, Isayama H, Sasaki T, Sasahira N, Tsujino T, Toda N, Kogure H, Matsubara S, Ito Y, Togawa O, Arizumi T, Hirano K, Tada M, Omata M, Koike K (2012) A multicentre randomised phase II trial of gemcitabine alone vs gemcitabine and S-1 combination therapy in advanced pancreatic cancer: GEMSAP study. Br J Cancer 106:1934–1939. 10.1038/bjc.2012.183 22555398 10.1038/bjc.2012.183 PMC3388559

[32] Ozaka M, Matsumura Y, Ishii H, Omuro Y, Itoi T, Mouri H, Hanada K, Kimura Y, Maetani I, Okabe Y, Tani M, Ikeda T, Hijioka S, Watanabe R, Ohoka S, Hirose Y, Suyama M, Egawa N, Sofuni A, Ikari T, Nakajima T (2012) Randomized phase II study of gemcitabine and S-1 combination versus gemcitabine alone in the treatment of unresectable advanced pancreatic cancer (Japan Clinical Cancer Research Organization PC-01 study). Cancer Chemother Pharmacol 69:1197–1204. 10.1007/s00280-012-1822-1 22249272 10.1007/s00280-012-1822-1

[33] Park J-W, Kim YJ, Kim DY, Bae S-H, Paik SW, Lee Y-J, Kim HY, Lee HC, Han SY, Cheong JY, Kwon OS, Yeon JE, Kim BH, Hwang J (2019) Sorafenib with or without concurrent transarterial chemoembolization in patients with advanced hepatocellular carcinoma: the phase III STAH trial. J Hepatol 70:684–691. 10.1016/j.jhep.2018.11.029 30529387 10.1016/j.jhep.2018.11.029

[34] Qin S, Bi F, Gu S, Bai Y, Chen Z, Wang Z, Ying J, Lu Y, Meng Z, Pan H, Yang P, Zhang H, Chen X, Xu A, Cui C, Zhu B, Wu J, Xin X, Wang J, Shan J, Chen J, Zheng Z, Xu L, Wen X, You Z, Ren Z, Liu X, Qiu M, Wu L, Chen F (2021) Donafenib versus sorafenib in first-line treatment of unresectable or metastatic hepatocellular carcinoma: a randomized, open-label, parallel-controlled phase II-III trial. J Clin Oncol Off J Am Soc Clin Oncol 39:3002–3011. 10.1200/JCO.21.00163 10.1200/JCO.21.00163 PMC8445562 34185551

[35] Kudo M, Ueshima K, Yokosuka O, Ogasawara S, Obi S, Izumi N, Aikata H, Nagano H, Hatano E, Sasaki Y, Hino K, Kumada T, Yamamoto K, Imai Y, Iwadou S, Ogawa C, Okusaka T, Kanai F, Akazawa K, Yoshimura K-I, Johnson P, Arai Y (2018) SILIUS study group: Sorafenib plus low-dose cisplatin and fluorouracil hepatic arterial infusion chemotherapy versus sorafenib alone in patients with advanced hepatocellular carcinoma (SILIUS): a randomised, open label, phase 3 trial. Lancet Gastroenterol Hepatol 3:424–432. 10.1016/S2468-1253(18)30078-5 29631810 10.1016/S2468-1253(18)30078-5

[36] Lyu N, Wang X, Li J-B, Lai J-F, Chen Q-F, Li S-L, Deng H-J, He M, Mu L-W, Zhao M (2022) Arterial chemotherapy of oxaliplatin plus fluorouracil versus sorafenib in advanced hepatocellular carcinoma: a biomolecular exploratory, randomized, phase III trial (FOHAIC-1). J Clin Oncol Off J Am Soc Clin Oncol 40:468–480. 10.1200/JCO.21.01963 10.1200/JCO.21.01963 34905388

[37] GEMZAR FDA AccessData https://www.accessdata.fda.gov/drugsatfda_docs/label/2011/020509s069lbl.pdf

[38] Bruix J, Cheng A-L, Meinhardt G, Nakajima K, De Sanctis Y, Llovet J (2017) Prognostic factors and predictors of sorafenib benefit in patients with hepatocellular carcinoma: analysis of two phase III studies. J Hepatol 67:999–1008. 10.1016/j.jhep.2017.06.026 28687477 10.1016/j.jhep.2017.06.026

[39] Llovet JM, Ricci S, Mazzaferro V, Hilgard P, Gane E, Blanc J-F, de Oliveira AC, Santoro A, Raoul J-L, Forner A, Schwartz M, Porta C, Zeuzem S, Bolondi L, Greten TF, Galle PR, Seitz J-F, Borbath I, Häussinger D, Giannaris T, Shan M, Moscovici M, Voliotis D, Bruix J (2008) SHARP investigators study group: sorafenib in advanced hepatocellular carcinoma. N Engl J Med 359:378–390. 10.1056/NEJMoa0708857 18650514 10.1056/NEJMoa0708857

[40] Sorafenib https://www.ema.europa.eu/en/documents/product-information/nexavar-epar-product-information_en.pdf

[41] Garcia-Cremades M, Pitou C, Iversen PW, Troconiz IF (2018) Predicting tumour growth and its impact on survival in gemcitabine-treated patients with advanced pancreatic cancer. Eur J Pharm Sci Off J Eur Fed Pharm Sci 115:296–303. 10.1016/j.ejps.2018.01.033 10.1016/j.ejps.2018.01.033 29366960

[42] Veerman G, Ruiz van Haperen VW, Vermorken JB, Noordhuis P, Braakhuis BJ, Pinedo HM, Peters GJ (1996) Antitumor activity of prolonged as compared with bolus administration of 2’,2’-difluorodeoxycytidine in vivo against murine colon tumors. Cancer Chemother Pharmacol 38:335–342. 10.1007/s002800050492 8674156 10.1007/s002800050492

[43] Choi YH, Zhang C, Liu Z, Tu M-J, Yu A-X, Yu A-M (2021) A novel integrated pharmacokinetic-pharmacodynamic model to evaluate combination therapy and determine in vivo synergism. J Pharmacol Exp Ther 377:305–315. 10.1124/jpet.121.000584 33712506 10.1124/jpet.121.000584 PMC8140393

[44] Zhang L, Sinha V, Forgue ST, Callies S, Ni L, Peck R, Allerheiligen SRB (2006) Model-based drug development: the road to quantitative pharmacology. J Pharmacokinet Pharmacodyn 33:369–393. 10.1007/s10928-006-9010-8 16770528 10.1007/s10928-006-9010-8

[45] Jain L, Woo S, Gardner ER, Dahut WL, Kohn EC, Kummar S, Mould DR, Giaccone G, Yarchoan R, Venitz J, Figg WD (2011) Population pharmacokinetic analysis of sorafenib in patients with solid tumours. Br J Clin Pharmacol 72:294–305. 10.1111/j.1365-2125.2011.03963.x 21392074 10.1111/j.1365-2125.2011.03963.x PMC3162659

[46] Matsumoto T, Masuo Y, Tanaka A, Kimura T, Ioroi T, Yamakawa T, Kitahara H, Kato Y (2022) A physiologically based pharmacokinetic and pharmacodynamic model for disposition of FF-10832. Int J Pharm 627:122250. 10.1016/j.ijpharm.2022.122250 36183917 10.1016/j.ijpharm.2022.122250

[47] Edginton AN, Zimmerman EI, Vasilyeva A, Baker SD, Panetta JC (2016) Sorafenib metabolism, transport, and enterohepatic recycling: physiologically based modeling and simulation in mice. Cancer Chemother Pharmacol 77:1039–1052. 10.1007/s00280-016-3018-6 27053087 10.1007/s00280-016-3018-6 PMC4846505

[48] Claret L, Girard P, Hoff PM, Van Cutsem E, Zuideveld KP, Jorga K, Fagerberg J, Bruno R (2009) Model-based prediction of phase III overall survival in colorectal cancer on the basis of phase II tumor dynamics. J Clin Oncol Off J Am Soc Clin Oncol 27:4103–4108. 10.1200/JCO.2008.21.0807 10.1200/JCO.2008.21.0807 19636014

[49] Schindler E, Amantea MA, Karlsson MO, Friberg LE (2017) A pharmacometric framework for axitinib exposure, efficacy, and safety in metastatic renal cell carcinoma patients. CPT Pharmacomet Syst Pharmacol 6:373–382. 10.1002/psp4.12193 10.1002/psp4.12193 PMC5488123 28378918

[50] Krishnan SM, Laarif SS, Bender BC, Quartino AL, Friberg LE (2021) Tumor growth inhibition modeling of individual lesion dynamics and interorgan variability in HER2-negative breast cancer patients treated with docetaxel. CPT Pharmacomet Syst Pharmacol 10:511–521. 10.1002/psp4.12629 10.1002/psp4.12629 PMC8129720 33818899

[51] Dogra P, Shinglot V, Ruiz-Ramírez J, Cave J, Butner JD, Schiavone C, Duda DG, Kaseb AO, Chung C, Koay EJ, Cristini V, Ozpolat B, Calin GA, Wang Z (2024) Translational modeling-based evidence for enhanced efficacy of standard-of-care drugs in combination with anti-microRNA-155 in non-small-cell lung cancer. MedRxiv Prepr Serv Health Sci. 10.1101/2024.03.14.24304306 10.1186/s12943-024-02060-5 PMC11295620 39095771

[52] Parab TM, DeRogatis MJ, Boaz AM, Grasso SA, Issack PS, Duarte DA, Urayeneza O, Vahdat S, Qiao J-H, Hinika GS (2019) Gastrointestinal stromal tumors: a comprehensive review. J Gastrointest Oncol 10:144–154. 10.21037/jgo.2018.08.20 30788170 10.21037/jgo.2018.08.20 PMC6351301

[53] Yoshino T, Shi Q, Misumi T, Bando H, Wakabayashi M, Raeisi M, Andre T, de Gramont A (2023) A synthetic control arm for refractory metastatic colorectal cancer: the no placebo initiative. Nat Med 29:2389–2390. 10.1038/s41591-023-02488-0 37507606 10.1038/s41591-023-02488-0

[54] Popat S, Liu SV, Scheuer N, Hsu GG, Lockhart A, Ramagopalan SV, Griesinger F, Subbiah V (2022) Addressing challenges with real-world synthetic control arms to demonstrate the comparative effectiveness of Pralsetinib in non-small cell lung cancer. Nat Commun 13:3500. 10.1038/s41467-022-30908-1 35715405 10.1038/s41467-022-30908-1 PMC9205915

[55] Yin X, Davi R, Lamont EB, Thaker PH, Bradley WH, Leath CA, Moore KM, Anwer K, Musso L, Borys N (2023) Historic clinical trial external control arm provides actionable GEN-1 efficacy estimate before a randomized trial. JCO Clin Cancer Inform 7:e2200103. 10.1200/CCI.22.00103 36608308 10.1200/CCI.22.00103

