# Supplementary material for: Predicting tumor dynamics in treated patients from patient-derived-xenograft mouse models: a translational model-based approach
Source: J Pharmacokinet Pharmacodyn. 2025 Apr 16;52(3):24. doi: 10.1007/s10928-025-09970-x (PMC12003590; doi:10.1007/s10928-025-09970-x)
Supplement: Supplementary file 2 — Supplementary file2 (PDF 614 kb) [file 10928_2025_9970_MOESM2_ESM.pdf]

Target journal: Journal of Pharmacokinetics and Pharmacodynamics

Predicting tumor dynamics in treated patients from patient-derived-xenograft mouse models: a translational model-based approach

Authors: D. Ronchi[1], E.M. Tosca[1], P. Magni[1]

---

1. Dipartimento di Ingegneria Industriale e dell'Informazione, Università degli Studi di Pavia, I-27100 Pavia, Italy

Corresponding author:
Paolo Magni paolo.magni@unipv.it

Supplementary Material S1

TGI data in PDX mice

Gemcitabine treatment of pancreatic cancer

Table S1 *Considered TGI studies in pancreatic cancer PDX mouse models. For each PDX mouse model, a single TGI study including one control arm and one Gemcitabine arm was available.*

PDX code*	Administration schedule	Route of administration	Dose	Patient ethnicity
PA0527	Q4D x 4	Intra-peritoneal	120	Asian
PA0692	Q3D x 21	Intra-peritoneal	60	Asian
PA1170	Day1,5/wk x 3.5wks	Intra-peritoneal	40	Asian
PA1178	Day1,4/wk x 3wks	Intra-peritoneal	40	Asian
PA1189	Day1,4/wk x 3.5wks	Intra-peritoneal	40	Asian
PA1194	Q3D x 7	Intra-peritoneal	60	Asian
PA1198	Q3D x 16	Intra-peritoneal	60	Asian
PA1266	Day1,4/wk x 3wks	Intra-peritoneal	40	Asian
PA1280	Q4D x 4	Intra-peritoneal	60	Asian
PA1301	Q3D x 14	Intra-peritoneal	120	Asian
PA1332	Q4D x 6	Intra-peritoneal	15	Asian
PA1338	Q3D x 21	Intra-peritoneal	60	Asian
PA1383	Day0,4,8,Day12,Day16,20,24,28	Intra-venous	60	Asian
PA1390	Day3,7,11,15	Intra-peritoneal	60	Asian
PA3029	Q3D x 9	Intra-peritoneal	120	Asian
PA3065	Q4D x 4	Intra-peritoneal	60	Asian
PA3137	Day1,4/wk x 3wks	Intra-peritoneal	40	Asian
PA3139	Day1,4/wk x 3wks	Intra-peritoneal	40	Asian
PA6259	Day1,4/wk x 3wks	Intra-peritoneal	60	Asian
PA6265	Day1,4/wk x 5wks	Intra-peritoneal	40	Asian
PA1168	Q3Dx6	Intra-peritoneal	100	Asian
PA1222	QW x 2wks	Intra-peritoneal	120	Asian
PA1233	Q4Dx4	Intra-peritoneal	60	Asian
PA1265	QW x 2wks	Intra-peritoneal	120	Asian
PA1644	Day1,4/wk x 2.5wks	Intra-peritoneal	40	Asian
PA3013	Day1,4/wk x 2wks	Intra-peritoneal	40	Asian
PA3126	Q4D x 6	Intra-peritoneal	15	Asian
PA3149	Day1,4/wk x 2wks	Intra-peritoneal	120	Asian
PA6233	QW x 2wks	Intra-peritoneal	120	Asian

Notes: PDX codes from HuBase database (Crownbio Bioscience Inc., https://www.crownbio.com/). List of abbreviations: QaD x b: dose administered once every a days for b times. Day X,Y/wk x Zwks: dose administered on days X and Y every week for Z weeks.

Sorafenib treatment of hepatocellular cancer

Table S2 *Considered TGI studies in hepatocellular cancer PDX mouse models. For each PDX mouse model, a single TGI study including one control arm and one Sorafenib arm was available.*

PDX code*	Administration schedule	Route of administration	Dose	Origin
LI0050	QDx12	Oral	50	Asian
LI0334	QDx50	Oral	50	Asian
LI0348	QDx21	Oral	50	Asian
LI0574	QDx14	Oral	50	Asian
LI0612	QDx12	Oral	60	Asian
LI0752	QDx17	Oral	50	Asian
LI0801	QDx22	Oral	50	Asian
LI0941	Day1-5/wk x 2wks	Oral	50	Asian
LI1005	QDx14	Oral	30	Asian
LI1025	QDx21	Oral	50	Asian
LI1035	QDx21	Oral	50	Asian
LI1054	QDx14	Oral	50	Asian
LI1057	QDx14	Oral	50	Asian
LI1068	QDx13	Oral	50	Asian
LI1069	QDx14	Oral	50	Asian
LI1074	QDx13	Oral	50	Asian
LI1078	QDx21	Oral	50	Asian
LI1081	QDx21	Oral	50	Asian
LI1088	QDx13	Oral	50	Asian
LI1097	QDx14	Oral	50	Asian
LI1098	QDx14	Oral	50	Asian
LI1646	QDx21	Oral	50	Asian
LI6206	QDx21	Oral	50	Asian
LI6664	QDx21	Oral	50	Asian

Notes: PDX codes from HuBase database (Crownbio Bioscience Inc., https://www.crownbio.com/). List of abbreviations: QaD x b: dose administered once every a days for b times. Day X,Y/wk x Zwks: dose administered on days X and Y every week for Z weeks.

Supplementary Material S2

Steps performed to reconstruct TTP curves:

1. Data Extraction:

   - Survival data for $S_{PFS}(t_d)$ and $S_{OS}(t_d)$ were digitized from referenced publications using *WebPlotDigitizer* (https://apps.automeris.io/wpd4/).
   - A precise time grid (t_d) was selected to ensure accuracy.

2. Interpolation of survival curves:

   - The digitized survival data were interpolated on a dense, evenly spaced time grid $t = \{0, 0.1, \dots, 24\}$ months.

3. Calculation of progression events:

   - At each time point t_i:
     i. The number of event-free patients (i.e., at risk for both progression and death) was calculated as:

     $$N_{EF}(t_i) = N \cdot S_{PFS}(t_i)$$

     ii. The number of patients who had died was computed as:

     $$N_D(t_i) = N \cdot (1 - S_{OS}(t_i))$$

     iii. The number of patients who had progressed (but not died) was determined by:

     $$N_P(t_i) = N - N_{EF}(t_i) - N_D(t_i)$$

     iv. The number of new progression events between two consecutive time points (t_i and t_{i-1}) was derived as:

     $$\max\left(0, N_P(t_i) - N_P(t_{i-1})\right)$$

4. Kaplan-Meier (KM) estimator application:

   - Using the progression events and the number of patients at risk ($N_{EF}(t_i)$), the Kaplan-Meier estimator was applied to reconstruct $S_{TTP}(t)$.

5. Confidence interval calculation:

   - The 5th ($L_P(t)$) and 95th percentile ($U_{TTP}(t)$) percentiles were calculated using the following formulas:

$$L_{TTP}(t) = S_{TTP}(t)^{e^{\frac{z_{0.05}}{\ln(S_{TTP}(t))} \cdot \frac{SE(t)}{S_{TTP}(t)}}}$$

$$U_{TTP}(t) = S_{TTP}(t)^{e^{\frac{-z_{0.05}}{\ln(S_{TTP}(t))} \cdot \frac{SE(t)}{S_{TTP}(t)}}}$$

where $z_{0.05}$ was the 5th percentile of the normal distribution and $SE(t) = \sqrt{S_{TTP}(t)(1 - S_{TTP}(t))/N}$ with N the size of patient cohort, approximated the standard error of $S_{TTP}(t)$.

Digitized survival curves are included as .xlsx file for transparency.

Supplementary Material S3

Pharmacokinetic compartment model of i.p. administration of Gemcitabine in mice.

Fig. S1 *Mice PK 2-compartment model with absorption: diagram.*

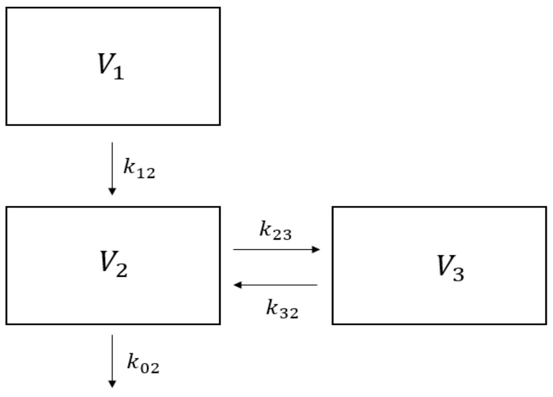

Table S3 *Mice PK model: parameters.*

Parameter	Value
$V_2[ml * kg^{-1}]$	887.2
$k_{12}[day^{-1}]$	92.272
$k_{32}[day^{-1}]$	20.376
$k_{23}[day^{-1}]$	45.192
$k_{02}[day^{-1}]$	177.12
$F[-]$	1

Pharmacokinetic compartment model of oral administration of Sorafenib in mice.

Table S4 *Mice PK model: parameters.*

Parameter	Value
$V_c[l]$	0.0112
$V_p[l]$	0.0120
$k_{12}[hour^{-1}]$	0.663
$k_{21}[hour^{-1}]$	0.620
$k_e[hour^{-1}]$	0.334
$k_a[hour^{-1}]$	0.640

Supplementary Material S4

Monte Carlo simulation procedure

For both the case studies, the following Monte Carlo simulation procedure was applied:

- to account for estimation uncertainty, parameters characterizing the $\lambda_0 - k_2$ log-normal distribution in mice, i.e., $\left(\lambda_{0,pop}, k_{2,pop}, \omega_{\lambda_0}, \omega_{k_2}, \omega_{\lambda_0,k_2}\right)$, were assumed to follow a multivariate normal distribution, $N(\boldsymbol{\mu},\boldsymbol{\Sigma})$, where $\boldsymbol{\mu}$ is the vector of parameter estimates obtained by identifying the Simeoni TGI model on PDX mice data and $\boldsymbol{\Sigma}$ the covariance matrix of the estimates;
- from the previous multivariate normal distribution, 1000 samples, $\left\{\lambda_{0,pop,j}, k_{2,pop,j}, \omega_{\lambda_0,j}, \omega_{k_2,j}, \omega_{\lambda_0,k_2,j}\right\}_{j=1\ldots1000}$ were extracted, thus obtaining 1000 $\lambda_0 - k_2$ log-normal distributions in mice;
- the 1000 $\lambda_0 - k_2$ log-normal distribution in mice were scaled to human according to Eq.5 of the main text, thus obtaining 1000 $\lambda_{0,human} - k_{2,human}$ log-normal distributions;
- from each of the $\lambda_{0,human} - k_{2,human}$log-normal distributions a cohort of N=200 virtual cancer patients were generated.

Overall, 1000 cohorts each composed by 200 virtual patients were generated accounting for parameter estimation uncertainty.

4.1 Predictive Intervals of Tumor growth

For each patient cohort, individual trajectories of tumor volume were simulated on a dense grid of time points and, then, converted in terms of tumor diameter using Eq.2. The median, the 5th and 95th percentiles $(p^{0.05}, p^{0.5}, p^{0.95})$ of tumor diameter trajectories among the 200 virtual patients were computed. Finally, the median and the 90% confidence interval (90%CI) of $p^{0.05}, p^{0.5}, p^{0.95}$ among the 1000 replicates in the 1000 virtual patients cohorts were considered.

4.2 Predictive Intervals of KM curves

For each virtual patient in each of the 1000 patient cohorts, the time to PFS events (i.e., PD or death which occurs first) was derived as detailed in the main text. Then, for each patient cohort, the KM estimator of PFS probability, $S(t)$, was computed together with its 90%CI, i.e., $\left(L(t),\ U(t)\right)$ which defined as:

$$L(t) = S(t)^{e^{\frac{z_{0.05}}{\ln(S(t))} \cdot \frac{SE(t)}{S(t)}}} \quad \text{and} \quad U(t) = S(t)^{e^{\frac{-z_{0.05}}{\ln(S(t))} \cdot \frac{SE(t)}{S(t)}}}$$

where $z_{0.05}$ was the 5th percentile of the normal distribution and $SE(t) = \sqrt{S(t)(1-S(t))/N}$ with N=200 the size of patient cohort, approximated the standard error of $S(t)$.
In this way, 1000 PFS curves, one for each patient cohort, were obtained. The median of $S(t)$, the 5th percentile of $L(t)$ and the 95th percentile of $U(t)$ among the 1000 patient cohorts, were finally considered.

Supplementary Material S5

Gemcitabine treatment of pancreatic cancer.

<u>Identification of the Simeoni TGI model.</u>
Random effects accounting for inter-PDX variability was introduced on all the parameters. Correlation was included only between random effect of λ_0 and k_2. A proportional error model was adopted. In the current case study, the initial tumor size is referred to the tumor volume at the time of the tumor implantation.

Table S5 *Obtained estimates of model parameters.*

**Parameter**	**Value**	**R.S.E. (%)**
$\lambda_0[day^{-1}]$	0.1	11.4
$\lambda_1[cm^3 \cdot day^{-1}]$	0.037	16.2
$TV_0\ [cm^3]$	0.0069	26.0
$k_1[day^{-1}]$	0.21	46.4
$k_2[L \cdot mg^{-1} \cdot day^{-1}]$	0.56	32.4
ω_{λ_0}	0.61	16.6
ω_{λ_1}	0.8	14.4
ω_{TV_0}	1.23	14.7
ω_{k_1}	1.94	24.4
ω_{k_2}	1.55	19.0
ρ_{k_2,λ_0} *	0.44	64.1
a	0.0036	21.4
b	0.13	4.15

*Notes: *$\rho_{k_2,\lambda_0} = \omega_{\lambda_0,k_2}/(\omega_{\lambda_0} \cdot \omega_{k_2})$ is the correlation between the random effects of λ_0 and k_2.*

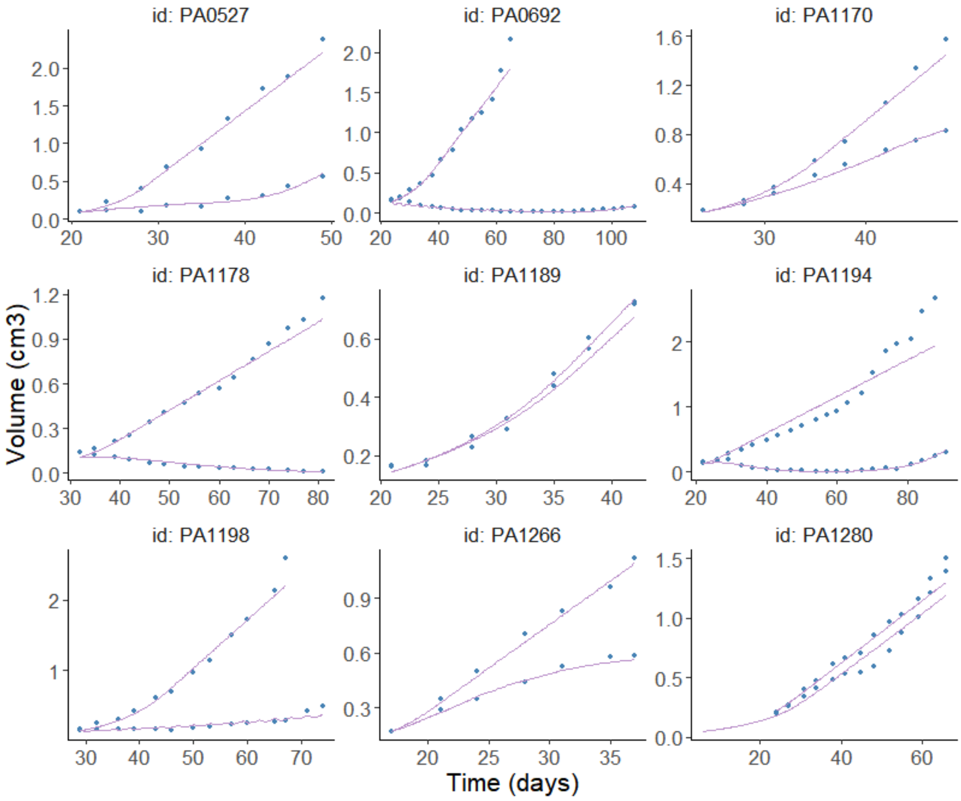

Fig. S2 *Individual fit plots for a subset of 9 TGI studies in 9 PDX mouse models of pancreatic cancer. In each panel, both the control arm and the Gemcitabine one are shown.*

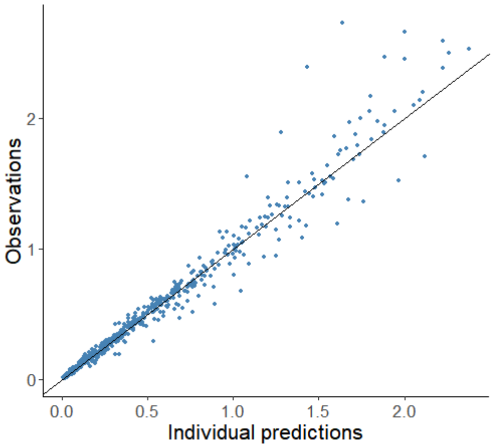

Fig. S3 *Individual Prediction vs Observations.*

Sorafenib treatment of hepatocellular cancer

Simeoni TGI model fit. The interindividual parameters have been introduced on all the parameters and a proportional error model has been adopted. In the current case study, the initial tumor size is referred to the tumor volume at the time of the first measurement.

Table S6 *Obtained estimates of model parameters*

Parameter	Value	R.S.E. (%)
$\lambda_0[day^{-1}]$	0.11	3.7
$\lambda_1[cm^3 * day^{-1}]$	0.056	11.9
$TV_0\ [cm^3]$	0.18	4.0
$k_1[day^{-1}]$	2.17	33.8
$k_2[L * mg^{-1} * day^{-1}]$	0.0061	13.2
ω_{λ_0}	0.54	18.3
ω_{λ_1}	0.51	17.3
ω_{TV_0}	0.17	16.5
ω_{k_1}	1.01	28.1
ω_{k_2}	0.63	16.1
ρ_{k_2,λ_0}	0.87	6.8
b	0.092	3.8

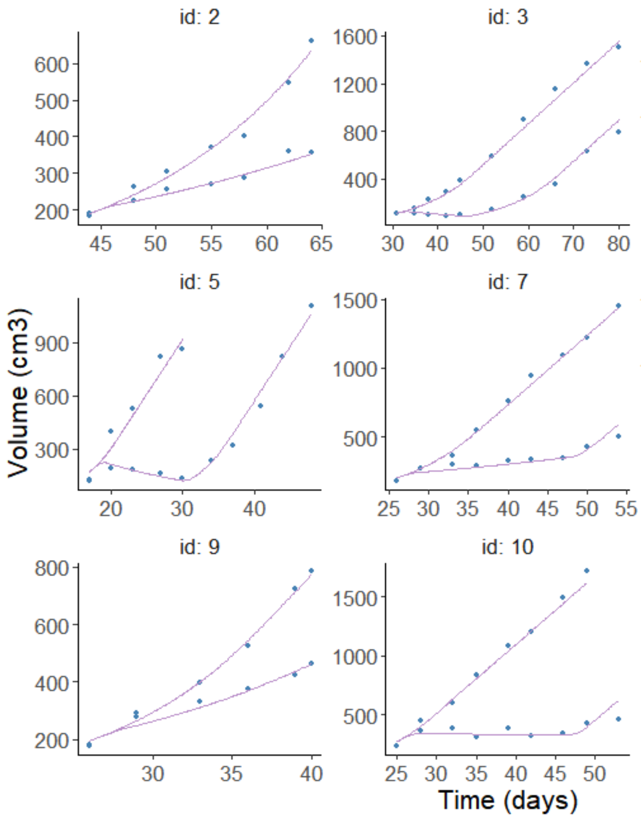

Fig. S4 *Individual fits for a subset of 9 PDX liver cancer model. In each panel, both the control arm and the Sorafenib one are shown.*

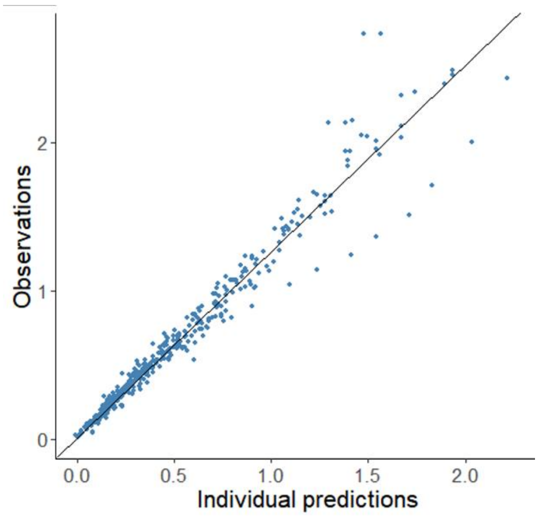

Fig. S5 *Individual Prediction vs Observations.*

Supplementary Material S6

<u>Hepatocellular cancer treated with Sorafenib</u>
Predicted tumor dynamics up to 14 months assuming strict adherence to nominal clinical protocol and $TV_0 = 1\ cm^3$ for all the virtual patients are reported below.

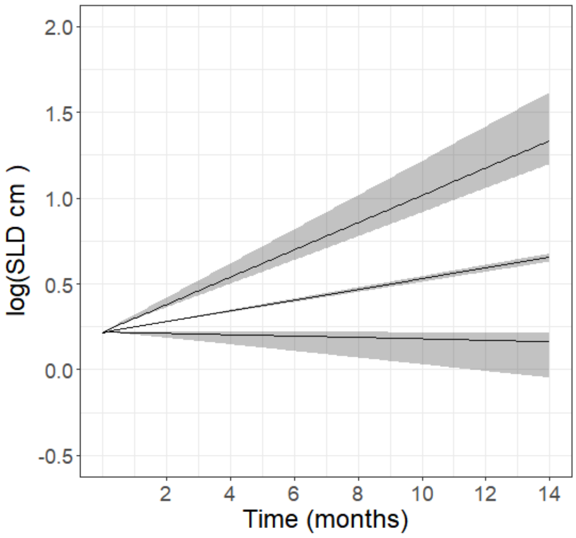

Fig. S8 *VPC of the simulated SLD trajectories over a 14-months period under the standard sorafenib treatment schedule. Solid lines represent the median and the 5th and 95th percentiles; dashed areas the corresponding 90%PI.*.
